# Narratives Argumentieren in politischen Leserbriefen

**DOI:** 10.1007/s41244-021-00200-8

**Published:** 2021-04-14

**Authors:** Juliane Schröter

**Affiliations:** Département de langue et de littérature allemandes, Université de Genève, Genf, Schweiz

**Keywords:** Argumentation, Argument, Narration, Erzählung, Politik, Leserbrief, Zeitung, Argumentation, Argument, Narrative, Storytelling, Politics, Letter to the Editor, Newspaper

## Abstract

Wie und warum werden Erzählungen in zeitgenössischen politischen Leserbriefen zur Argumentation verwendet? Um Antworten auf diese Frage zu gewinnen, untersuche ich gut 50 Leserbriefe aus Schweizer und deutschen Tageszeitungen, die alle einen Bezug zu COVID-19 haben. In allen Briefen wird argumentiert, indem erzählt wird. Es zeigt sich, dass die Narration in der Regel als Prämisse eines Arguments durch Induktion in einer Argumentation mit evaluativem Standpunkt fungiert. Eine solche Prämisse zu erzählen, hat für die Schreibenden den Vorteil, dass sie mit dem beispielhaften Geschehen sukzessive auch dessen Bewertung vermitteln können. Ein gesellschaftlich-kultureller Mehrwert solch narrativer Argumente durch Induktion lässt sich darin erkennen, dass sie in politischen Diskussionen auf unkomplizierte Weise die soziale Mikroebene mit der gesellschaftlichen Makroebene verbinden können.

## Abstract (en)

How and why are narratives used for argumentation in contemporary political letters to the editor? In order to find answers to this question, I examine about 50 letters to the editor from Swiss and German daily newspapers, all of which are related to COVID-19. In all letters, the writer presents an argument by telling a story. It turns out that the story usually serves as a premise for an argument from example in an argumentation with an evaluative standpoint. For the writers, unfolding such a premise as a narrative has the advantage that they can successively convey an evaluation together with the exemplary event. A socio-cultural benefit of such narrative arguments from example might be that they can easily connect the social micro-level with the societal macro-level in political debates.

## Einführung

»Der Leserbrief ist vorwiegend argumentativ-erörternd angelegt«, bemerkt Ulla Fix (2012, S. 142) in ihrer umfassenden Charakterisierung des Leserbriefs als Textsorte. Sie fügt hinzu, dass »auch deskriptive, partielle narrative Passagen […] vorhanden sein [können]«.

Diese knappe, nicht weiter ausgeführte Feststellung Fix’ bildet den Ausgangspunkt meines Beitrags. Allerdings gehe ich darin nicht nur davon aus, dass viele Leserbriefe zu politischen Gegenwartsfragen argumentative und narrative Abschnitte enthalten. Ich nehme auch an, dass die narrativen Abschnitte häufig der Argumentation dienen und dass sich zeitgenössische politische Leserbriefe deshalb sehr gut zur Beobachtung der Verbindung von Argumentieren und Erzählen in der politischen Kommunikation eignen. Die Frage, die ich hier verfolgen möchte, lautet dementsprechend: Wie und warum werden Erzählungen in zeitgenössischen politischen Leserbriefen aus deutschsprachigen Zeitungen zur Argumentation verwendet?

Auf diese Fragen hin untersuche ich Leserbriefe aus zwei deutschen und zwei Schweizer Tageszeitungen. Damit die Leserbriefe vergleichbar sind, haben sie alle einen thematischen Bezug zu COVID-19, der in der Alltagssprache oft auch als *Corona *oder *Coronavirus *bezeichneten Krankheit, die im Frühling 2020 als Pandemie auftrat. Mein Ziel ist es, am Beispiel von Leserbriefen verschiedene Formen und Funktionen zu erschließen, die Erzählen als Teil und zum Zweck des politischen Argumentierens annehmen kann.

## Forschung und zentrale Begriffe

Die relevante Forschung für meine Analyse betrifft vier Gegenstände: in erster Linie die Textsorte des Leserbriefs und die Verbindung von Argumentation und Narration, in zweiter Linie Argumentation und Narration bzw. Erzählen generell.

Leserbriefe sind seit vielen Jahren insbesondere in der Linguistik und Publizistikwissenschaft als Untersuchungsgegenstand etabliert. Die bisherigen Forschungsbeiträge sind allerdings insofern recht heterogen und nicht leicht vergleichbar, als sie Leserbriefe in verschiedenen Sprachen, aus unterschiedlichen geschichtlichen Phasen, in diversen Presseorganen und zu zahlreichen Themen betreffen. Zudem sind in der Forschung unterschiedliche analytische Akzente gesetzt worden: So sind Leserbriefe etwa mehrfach als Medien-Textsorte untersucht worden (z. B. Mémet 2005; Fix 2007; 2012; Burger/Luginbühl 2014), auf interkulturelle Differenzen hin befragt worden (z. B. Drewnowska-Vargáné 2001; Pounds 2005; Rojas-Lizana 2014) oder zum Gegenstand didaktischer Überlegungen gemacht worden (z. B. Menges/Wetekam 2011; Knoop 2019). Wichtig für meine Untersuchung sind Publikationen zu deutschsprachigen, gegenwartsnah entstandenen Leserbriefen, aus denen sich eine soziokulturell passende Begriffsbestimmung von *Leserbrief *entnehmen oder ableiten lässt. Unter Bezug auf Ulla Fix (2007, S. 215–223; 2012, S. 139–142), Julia Heupel (2007, S. 19–29) sowie Harald Burger und Martin Luginbühl (2014, S. 94, 100–102) fasse ich *Leserbriefe* hier als Texte auf, die von einer/einem Lesenden einer Zeitung oder Zeitschrift geschrieben und zur Publikation in einer Druckausgabe dieser Zeitung oder Zeitschrift an deren Redaktion gesandt worden sind. Leserbriefe wenden sich folglich an die Leserschaft eines Presseorgans, an dessen Redaktion und meist zugleich an eine umfassendere Öffentlichkeit, zu der auch diejenigen gehören, die für politische Entscheidungen verantwortlich sind. Die publizierten Leserbriefe sind von der jeweiligen Redaktion ausgewählt und anschließend häufig gekürzt oder umformuliert worden. Sie variieren relativ stark in der Länge und Komplexität – und zwar oft in deutlicher Abhängigkeit von der Zeitung oder Zeitschrift, in der sie erschienen sind. Leserbriefe betreffen normalerweise ein aktuelles Thema und erfolgen in der Regel als Reaktion auf einen oder mehrere Artikel, die in dem Blatt zu diesem Thema oder jedenfalls dem zugehörigen Themenbereich erschienen sind. Sie enthalten üblicherweise einen Standpunkt dazu. Daraus ergeben sich zwei Hauptfunktionen für den/die Schreibende: einerseits geht es in Leserbriefen darum, andere vom eigenen Standpunkt zu überzeugen; andererseits geht es oft (auch) darum, Affekte wie Empörung oder Begeisterung zum Ausdruck zu bringen. Ist man sich dieser Charakteristika der Textsorte bewusst, kann man gut nachvollziehen, warum Leserbriefe in der Forschung und in der Öffentlichkeit häufig als gut zugängliches Mittel der Einflussnahme auf die öffentliche Meinung und insofern als demokratisches Instrument der politischen Partizipation eingeschätzt werden. Zu den Textsorten, die der des Leserbriefs ähneln, aber von dieser abzugrenzen sind, zählen die Zuschrift zu einer Zeitungs- bzw. Zeitschriftenredaktion, die entweder nicht zur Veröffentlichung gedacht ist oder aber eine (z. B. medizinische oder psychologische) Beratungsanfrage enthält, und der Kommentar auf der Website einer Zeitung bzw. Zeitschrift (zu den Besonderheiten solcher Online-Kommentare und deren Unterschieden gegenüber ›klassischen‹ Leserbriefen vgl. z. B. Landert/Jucker 2011; Franz 2018).

Die Verbindung von Argumentation und Narration bildet den zweiten Gegenstand, der für diesen Beitrag unmittelbar von Bedeutung ist. Erst in den letzten Jahren sind vermehrt Forschungsbeiträge dazu erschienen. Unter Beteiligung von Forschenden aus diversen Disziplinen, etwa der Argumentationstheorie, Linguistik, Philosophie und Literaturwissenschaft, hat sich daraus zuletzt ein zusammenhängendes Forschungsfeld entwickelt. Für meine Studie relevant sind neben einführenden und überblicksartigen Beiträgen zur Verschränkung von Narration und Argumentation (z. B. Danblon 2008; Tindale 2017; Hannken-Illjes 2018, S. 153–163; Bleumer/Hannken-Illjes/Till 2019; Till 2019) vor allem Veröffentlichungen, die zeigen, wie Erzählungen in bzw. für Argumentationen verwendet werden können. Beschrieben worden ist ein solcher Einsatz in ganz unterschiedlichen Kontexten, wiederholt z. B. in der fiktionalen Literatur, beispielsweise in Fabeln, Parabeln und Romanen (etwa Hunt 2009; Govier/Ayers 2012; Plumer 2015; Green 2017), in juristischen Zusammenhängen (exemplarisch Hannken-Illjes 2006; Jonge 2008; Carranza 2015; Hannken-Illjes 2019) oder auch in der politischen Kommunikation (unter anderem Paparouni 2008; Sprain/Hughes 2015; Weidacher 2018; Girnth/Burggraf 2019). In der Zusammenschau der Beiträge zeichnet sich eine große formale Vielfalt der Erzählungen ab, die argumentativ verwendet werden: Solche Erzählungen können offenbar sowohl kurz, einfach und fragmentarisch als auch umfassend, komplex und geschlossen sein, sowohl fiktional als auch mit einem Faktizitätsanspruch verbunden sein, sowohl retrospektiv als auch zukunftsbezogen sein, sowohl allein stehen als auch von explizit formulierten Prämissen und/oder Konklusionen ergänzt werden, und solche Erzählungen können Argumente unterschiedlicher Typen bzw. Schemata bilden (z. B. Analogie- oder Induktionsargumente). Die argumentative Nutzung von Narrationen in Leserbriefen ist meines Wissens jedoch noch nicht untersucht worden. Diese ist insofern besonders interessant, als Erzählungen oder Berichte in dieser Textsorte – anders als z. B. in Fabeln, Parabeln oder Zeugenaussagen – keineswegs zwingend notwendig oder auch nur hochgradig erwartbar sind. Stärker als viele der bisher untersuchten Gattungen und Textsorten legen Leserbriefe deshalb die Frage nahe, warum im Rahmen einer Argumentation überhaupt erzählt wird oder, anders formuliert, welchen funktionalen Mehrwert Narrationen für Argumentationen bieten. Doch nicht nur durch den Gegenstand der Leserbriefe und durch die Frage nach dem funktionalen Mehrwert von Narrationen für Argumentationen geht die hier vorgestellte Untersuchung neue Wege. Auch die empirische Analyse seriellen Quellenmaterials unterscheidet sie von den meisten bisherigen Publikationen zur Verbindung von Argumentation und Narration.

Ein Überblick über die Forschung zu Argumentation und zu Narration generell kann hier natürlich nicht gegeben werden. Allerdings ist es notwendig, unter Bezug auf die Forschungsliteratur zu Arbeitsdefinitionen beider Konzepte zu gelangen. Diese sollen bei der Analyse der Leserbriefe eine möglichst klare Entscheidung darüber ermöglichen, ob und wo ggf. argumentiert bzw. erzählt wird. Der Begriff der *Argumentation* erscheint in der Forschungsliteratur als ein vergleichsweise unstrittiger, auch wenn in den jeweiligen Erläuterungen des Begriffs durchaus unterschiedliche Schwerpunkte gesetzt werden (vgl. exemplarisch Perelman/Olbrechts-Tyteca 1971, S. 4; Amossy 2006, S. 37; Eemeren et al. 2014, S. 7). Als knappe, aber konsensfähige Definition erscheint diejenige von Kati Hannken-Illjes (2018, S. 20): »Argumentation ist die Bearbeitung einer Streitfrage durch das Geben und Nehmen von Gründen«. Im Anschluss daran und unter Einbezug weiterer häufig genannter Bestimmungsmomente wird *Argumentation* hier verstanden als eine primär sprachliche Praktik als Prozess (den ich auch *Argumentieren* nenne) und Produkt, die auf eine Überwindung oder Verringerung des Zweifels an einem Standpunkt oder der Verschiedenheit von Standpunkten zielt. Eine Argumentation besteht aus mindestens einem argumentativen Schluss (der hier ebenfalls als *Argument *bezeichnet wird), und dieser setzt sich aus einem Set von Prämissen (die im Folgenden auch *Gründe* genannt werden) und einer Konklusion zusammen. Mindestens eine der Prämissen muss explizit formuliert werden, die weiteren Prämissen und sogar die Konklusion können hingegen implizit bleiben (für diese Definition vgl. Schröter 2019, S. 298; Schröter/Thome 2020, S. 265; Schröter 2021, S. 1–3).

Die Begriffe der *Narration *bzw. *Erzählung *sind demgegenüber strittiger. Das hat auch damit zu tun, dass im Deutschen zusätzlich die Ausdrücke *Geschichte *und *Narrativ* existieren, deren Bedeutungsspektren sich mit denjenigen von *Erzählung *und *Narration* überlappen. Auf Englisch und Französisch wiederum wird der Bedeutungsbereich der genannten Ausdrücke von anderen Wörtern (*narration, story, fiction, plot, narrative, narration, histoire, conte, récit, intrigue* etc.) anders aufgeteilt. Trotz dieser Unübersichtlichkeit lässt sich festhalten, dass *Erzählung* in der germanistischen Literatur- und Sprachwissenschaft erstens für eine einzelne epische Gattung verwendet wird, zweitens als Oberbegriff für verschiedene epische Gattungen und drittens in einer weiteren Bedeutung, um die es hier geht. Diese weitere Bedeutung bringt Matías Martínez treffend auf die Formel »Geschehensdarstellung + x«, wobei mit *x *eines oder mehrere weitere Merkmale wie »Kausalität«, »Ganzheit« oder »Tellability« gemeint sind, die für sich genommen zwar typisch, aber nicht notwendig für Erzählungen sind (Martínez 2017, S. 3–5, vgl. 3–6; zu den drei Bedeutungen vgl. auch Schmeling/Walstra 2007, S. 517–518). Zusätzlich wird *Erzählung*, insbesondere aber *Grosserzählung*, mitunter für *Narrativ *gebraucht. Während *Erzählung* in literatur- und sprachwissenschaftlichen Nachschlagewerken ein übliches Lemma ist, bildet *Narration *viel seltener ein solches und ist dementsprechend (noch) weniger klar definiert. In diesem Beitrag wird *Narration *gleichbedeutend mit *Erzählung *in der dritten genannten Lesart verwendet. Unter Bezug auf Martínez Vorschlag, aber auch auf die umfangreiche linguistische Forschungsdiskussion über Alltagserzählungen (vgl. z. B. Gülich/Hausendorf 2000; Becker/Quasthoff 2005; Georgakopoulou 2007; Spieß/Tophinke 2018) verstehe ich hier unter *Narration *eine primär sprachliche Praktik als Prozess (den ich auch *Erzählen* nenne) und Produkt (das ich ebenfalls als *Erzählung *bezeichne), die auf die Vermittlung eines Geschehens zielt. Sie besteht aus der Darstellung mindestens zweier zeitlich aufeinanderfolgender Zustände derselben menschlichen oder anthropomorphen Entität. Die dargestellte Zustandsveränderung wird kommunikativ interessant gemacht, was mit sehr unterschiedlichen Mitteln möglich ist, z. B. durch eine anschauliche, detailreiche, spannungserzeugende, perspektivisch-emotionale Darstellungsweise, durch eine metakommunikative Einführung als erzählenswert, aber auch etwa durch eine Bezeichnung oder kontextuelle ›Aufmachung‹ als Roman, Novelle oder Kurzgeschichte.

## Korpus und Methodik

Das Korpus, das meiner Untersuchung zugrunde liegt, umfasst etwas mehr als 50 Leserbriefe. Je zehn bis zwölf Briefe stammen aus vier auflagenstarken deutschsprachigen Tageszeitungen, die unterschiedlichen Medienunternehmen gehören (vgl. IVW 2020; WEMF 2020):

- aus einer Schweizer Qualitätszeitung, dem *Tages-Anzeiger *(TA),
- einer deutschen Qualitätszeitung, der *Süddeutschen Zeitung *(SZ),
- einer Schweizer Boulevardzeitung, dem *Blick*, und
- einer deutschen Boulevardzeitung, der *B.Z.*

Darüber hinaus enthält das Korpus einige Zufallsfunde aus weiteren deutschen und Schweizer Zeitungen.

Alle einbezogenen Leserbriefe haben einen thematischen Bezug zu *COVID-19*, zur alltagssprachlich auch *Corona *oder *Coronavirus *genannten Krankheit, die durch das Coronavirus SARS-CoV‑2 verursacht wird, und zum politisch-gesellschaftlichen Umgang damit. Die Leserbriefe berühren somit ein Problem, das eine politische Dimension hat, insofern Regierungen und Parlamente ihm mit politischen Maßnahmen beizukommen versuchen (ich schließe diesen Beitrag im Herbst 2020 während der sogenannten ›zweiten Welle‹ der Pandemie in Europa ab). Zugleich und im Kern ist das Problem jedoch ein medizinisch-biologisches, das sich in der Schweiz und in Deutschland grundsätzlich identisch verhält. Texte mit Bezug zu COVID-19 sind insofern für eine länderübergreifende Untersuchung besonders geeignet. Alle untersuchten Leserbriefe sind im ersten Halbjahr 2020 erschienen, als die COVID-19-Pandemie ihren ersten Höhepunkt in Europa erreichte und sowohl in den Schweizer als auch in den deutschen Medien überaus präsent war.

Die meisten Leserbriefe des Korpus wurden über Zeitungs-Datenbanken gefunden. Ich habe dort nach Artikeln der vier genannten Zeitungen gesucht, die die Ausdrücke Leser* und zudem Corona* oder COVID* enthalten. Die Artikel, die auf diese Suchanfrage hin angezeigt wurden, habe ich einzeln durchgesehen. Ins Korpus aufgenommen wurden nur Leserbriefe, die nach den obigen Arbeitsdefinitionen Argumentation und Narration enthalten. In allen aufgenommenen Leserbriefen wird argumentiert, indem (unter anderem) erzählt wird. Dabei habe ich darauf geachtet, dass die ausgewählten Leserbriefe innerhalb des Themenbereichs *COVID-19 *möglichst unterschiedliche thematische Schwerpunkte setzen und ein breites stilistisches Spektrum abdecken. Die Themenschwerpunkte, die je zwei oder mehr Leserbriefe setzen, sind Medizin/Gesundheitswesen, Reise/Verkehr, Einzelhandel/Banken, Wirtschaft/finanzielle Hilfe, Presse/Medien, Polizei/Behörden, Kinderbetreuung/(Aus)Bildungswesen sowie öffentlicher Raum/Veranstaltungen.

Die ausgewählten Leserbriefe habe ich anschließend auf verschiedene formale und funktionale Merkmale hin untersucht, und zwar daraufhin,

- wie lang die enthaltene Narration ist und wie groß ihr Anteil am Gesamttext ist,
- welche Rolle die Narration für die Argumentation übernimmt (z. B. die Rolle einer Prämisse oder Konklusion) und wie explizit das jeweilige narrative Argument ist (d. h. der argumentative Schluss, zu dem die Narration beiträgt),
- auf welches abstraktere Argumentationsschema das narrative Argument zurückzuführen ist (z. B. Argument über ein Analogieverhältnis, Argument durch Induktion usw.) und welche Art von Standpunkt es stützt (deskriptiver, evaluativer oder präskriptiver Standpunkt) sowie
- welche Formulierungsbesonderheiten der Standpunkt und die Narration aufweisen.

Die Analyse wurde mit größerem zeitlichem Abstand doppelt durchgeführt. Dabei wurde der Wortlaut der Leserbriefe möglichst genau beachtet. Alle verbleibenden Zweifelsfälle wurden gleich klassifiziert.

Bei der folgenden Darstellung der Untersuchungsergebnisse geht es mir in erster Linie darum, die formale und funktionale Vielfalt der argumentativen Narrationen aufzuzeigen. Trotzdem gebe ich auch Hinweise darauf, welche Formen bzw. Funktionen wie häufig vorkommen. Nicht im Vordergrund stehen im Folgenden die Unterschiede zwischen den Schweizer und den deutschen, den Qualitäts- und den Boulevardzeitungen. Diese können aufgrund der kleinen Zahl von Leserbriefen aus jeder der vier genannten Zeitungen nicht zuverlässig ermittelt werden. Besonders auffällige Differenzen werden gleichwohl erwähnt.

## Ergebnisse

### Länge der Narration und Anteil der Narration am Gesamttext

Abgesehen von einem Zweifelsfall enthalten alle Leserbriefe genau eine Narration, in der die/der Schreibende fast immer von eigenen Erlebnissen und Erfahrungen erzählt. Wie lang die Narration sein kann, hängt selbstverständlich von der Länge der Leserbriefe ab. Die Länge der Leserbriefe des Korpus variiert deutlich, und zwar auffallend stark in Abhängigkeit von der Zeitung, in der sie erschienen sind. Gibt man die Länge der Leserbriefe ohne allfällige Überschrift und Urheberidentifizierung an, beträgt sie

- im *Tages-Anzeiger* durchschnittlich 117 Wörter (min. 53 und max. 180 Wörter),
- in der *Süddeutschen Zeitung* durchschnittlich 246 Wörter (min. 66 und max. 377 Wörter),
- im *Blick *durchschnittlich 45 Wörter (min. 25 und max. 60 Wörter) und
- in der *B.Z.* durchschnittlich 54 Wörter (min. 37 und max. 69 Wörter).

Die Leserbriefe der Boulevardzeitungen sind somit im Durchschnitt deutlich kürzer als die der Qualitätszeitungen, wobei die der Schweizer Zeitungen im Durchschnitt kürzer sind als die der deutschen Zeitungen.

Der Kürze vieler Leserbriefe entsprechend, findet sich darin oftmals eine »small story« (Georgakopoulou 2007, S. 2, 147–148), eine Mikroerzählung, die von prototypischen literarischen oder biographischen Erzählungen allein deshalb relativ stark abweicht, weil sie kurz bis sehr kurz ausfällt. Ein Beispiel für eine sehr kurze Erzählung ist:

1. Meine Arbeitskollegin ist vor Sonntag von Mailand zurückgekommen, mit ihrem Sohn, der hohes Fieber hatte. Niemand hat sich dafür interessiert. Zum Glück wurde er vorher bereits genau untersucht. (Caduff 2020, Blick)

Trotz der Kürze des Zitats finden sich darin alle notwendigen Merkmale einer Erzählung im oben angegebenen Verständnis: So werden mehrere, zeitlich aufeinanderfolgende Zustände bzw. Geschehensetappen dargestellt, die Mutter und Sohn erlebt haben (Fieber – Untersuchung in Italien – Heimreise – Desinteresse in der Schweiz). Die dargestellten Zustände werden zudem mit dem intensivierenden Adjektiv *hohes*, der übertreibenden Formulierung *niemand hat sich dafür interessiert *und dem bewertenden Phraseologismus *zum Glück *interessant gemacht. Diese sprachlichen Mittel vergrößern den Kontrast zwischen dem Erwartbaren (Untersuchung in der Schweiz) und dem tatsächlich Eingetretenen (Desinteresse der Schweizer Ärzte), und sie bewerten das Vorgehen des medizinischen Personals in der Schweiz indirekt negativ. Aufgrund des Kontextes lässt sich das Zitat als Prämisse für die Konklusion *COVID-19-Verdachtsfälle werden in der Schweiz nicht ernst genug genommen *auffassen.

Am anderen Ende des beobachtbaren Längenspektrums befindet sich die Erzählung eines Leserbriefs aus der *Zeit*, der insgesamt 942 Wörter umfasst. In dem Leserbrief erzählt eine schwangere Frau mit zahlreichen Details und spannungserzeugenden Wendungen über viele Absätze hinweg, wie nur sie kurz vor der Geburt ihres Kindes noch von Ecuador nach Deutschland habe einreisen können, nicht aber ihr Mann, der keinen deutschen Pass besitze und deshalb nun wohl die Geburt des Kindes verpasse. Die Erzählung stützt eine Konklusion, die man mit *Die plötzlichen Änderungen der Einreisebestimmungen und des Flugverkehrs nach Deutschland aufgrund von COVID-19 sind abzulehnen* wiedergeben kann. In den von mir untersuchten und gesichteten Leserbriefen ist diese Narration allerdings mit Abstand die umfangreichste.

Neben der absoluten Länge der Erzählung variiert auch deren relative Länge stark, womit der Anteil der narrativen Abschnitte am Gesamttext gemeint ist. In den untersuchten Leserbriefen werden die narrativen Abschnitte – die für die weitere Analyse identifiziert und markiert wurden – normalerweise von anderen, oft rein argumentierenden Passagen ergänzt. In den längeren Leserbriefen aus den beiden Qualitätszeitungen erhalten diese anderen Passagen insgesamt mehr Raum als in den Leserbriefen der Boulevardzeitungen, weshalb unter den Leserbriefen der Qualitätszeitungen auch die mit den meisten Argumenten sind. Zur Illustration kann ein Leserbrief dienen, in dem ein Paar erzählt, wie seine vierköpfige Familie zu Hause Karten gespielt habe und dabei von der Polizei unterbrochen worden sei, die eine Buße wegen Nicht-Beachtung der COVID-19-Regeln angedroht habe. Der Text enthält jedoch auch größere zweifelsfrei nicht-narrative Teile wie den folgenden, abschließenden Abschnitt. In ihm werden für den Standpunkt des Gesamttextes – *Die derzeitigen weitreichenden Maßnahmen gegen COVID-19 in Bayern sind abzulehnen* – sowohl verschiedene Gründe als auch ein Gegengrund aufgeführt. Dies ist unter anderem an den lexikalischen und phraseologischen Argumentationsindikatoren *steht außer Frage, aber, wirklich, doch, um *und* sonst *erkennbar:

(2) Dass zur Bekämpfung des Coronavirus außergewöhnliche Maßnahmen erforderlich sind, steht außer Frage. Aber rechtfertigt das wirklich jede Maßnahme? Die Gefahren sind doch von Großveranstaltungen ausgegangen (Karneval in Heinsberg, Champions-League-Spiel von Atalanta Bergamo, Skibar in Ischgl), und nicht von Treffen im Familienkreis. Die bayerische Politik sollte die Einschränkungen der Grundrechte schleunigst auf ein realistisches Maß zurückfahren, um die Verhältnismäßigkeit sicherzustellen (zulässig ist es nur, das mildeste Mittel der Grundrechtseinschränkungen zu wählen). Die Privatsphäre der Menschen muss respektiert werden. Sonst werden große Teile der Bevölkerung kriminalisiert, der soziale Frieden gefährdet, und die Zweifel an der Sinnhaftigkeit der Maßnahmen steigen. (Mayer-Schwender [sic]/Schwendner 2020, SZ)

Wie im hier zitierten Leserbrief wird die Narration in der Regel von nicht-narrativen Textteilen gefolgt oder eingeführt oder auch gerahmt. Ab und zu wird sie allerdings auch von nicht-narrativen Abschnitten durchbrochen.

Einige wenige untersuchte Leserbriefe bestehen hingegen – jedenfalls in der veröffentlichten Fassung – ausschließlich aus der Narration, sieht man von der Verfasseridentifikation und ggf. auch der Überschrift ab. Ein Beispiel dafür ist die ausführliche Schilderung einer Reise von Bangkok nach Landshut, aus der hervorgeht, wie unterschiedlich und inkonsequent Flughäfen, Fluggesellschaften und Behörden COVID-19-Schutzmaßnahmen für Reisende umsetzen. Der Anfang und das Ende des Leserbriefs lauten:

(3) 30. April: Flughafen Bangkok. Zahlreiche Flughafenmitarbeiter achten schutzbekleidet auf Abstandsregeln beim Einchecken. Vor der Passkontrolle wird Fieber gemessen, überall sind Handdesinfektionsmöglichkeiten, am Abflug-Gate ist ausreichend Platz, um Abstand zu halten. Flug nach Amsterdam. Die Maschine ist voll, ich habe im Mittelgang einen freien Platz rechts von mir. Abstandsregeln und Hygienevorschriften sind schwer umsetzbar. […]
  4. Mai: Landshut. Ich muss mich (siehe Merkblatt) unverzüglich beim Gesundheitsamt melden. Laut Internet hat es geöffnet. Ich fahre hin und lese am verschlossenen Eingang die Aufforderung zur Terminvereinbarung. Um 14.19 Uhr schicke ich eine Email mit Terminwunsch. Bisher keine Antwort. Ich mache jetzt mal die zwei Wochen Quarantäne fertig und warte. (Langwieser 2020, SZ)

Die so beginnende und endende Narration wird nicht von nicht-narrativen Passagen unterbrochen. Dies wirft die Frage auf, inwiefern in Briefen wie diesem überhaupt argumentiert wird.

### Rolle der Narration für die Argumentation und Explizitheit des narrativen Arguments

Wie kann man behaupten, dass die Erzählung, die in einem Leserbrief enthalten ist, der Argumentation dient, wenn der Leserbrief aus nichts anderem als der Erzählung besteht? Die Antwort auf die Frage hat vor allem mit der Textsorte des Leserbriefs zu tun. Da Leserbriefe, wie in Abschnitt 2 erwähnt, normalerweise einen Standpunkt enthalten, kann die Textsorte als kontextueller Hinweis darauf gewertet werden, dass eine Argumentation vorliegt. Die Textsorte legt die Suche nach einem Standpunkt und einem oder mehreren Argumenten dafür nahe. Wie oben angesprochen, muss jedoch die Konklusion eines Arguments und also auch der Standpunkt einer Argumentation (der ja nichts anderes ist als die ranghöchste Konklusion) nicht unbedingt explizit sein. Im zuletzt zitierten Beispiel führt die Suche nach einem Standpunkt und einem oder mehreren Argumenten schnell zum Erfolg: Die Erzählung lässt sich als komplexe explizite Prämisse verstehen, die man mit den Worten paraphrasieren könnte: *Die COVID-19-Schutzmaßnahmen der von mir erlebten Flughäfen, Fluggesellschaften und Behörden sind widersprüchlich*. Diese Prämisse wird narrativ etabliert. Daraus wiederum lässt sich als implizite Konklusion erschließen: *Die COVID-19-Schutzmaßnahmen der Flughäfen, Fluggesellschaften und Behörden generell sind widersprüchlich*. Da es sich um eine einfache Argumentation handelt, die nur aus einem argumentativen Schluss besteht, bildet die Konklusion zugleich den Standpunkt.

In einigen wenigen Leserbriefen kann man die Narration folglich mit gutem Grund als explizite Prämisse für den impliziten Standpunkt auffassen. Viel öfter übernimmt die Narration in den untersuchten Leserbriefen jedoch die Rolle einer expliziten Prämisse für den expliziten Standpunkt. Dies lässt sich etwa in der nächsten kurzen Leserzuschrift beobachten:

(4) Mein Arzt erhält für das Ausfüllen eines einseitigen Datenblatts 60 Franken von meiner Versicherung. Der zeitliche Aufwand dafür beträgt maximal 5 Minuten. Wenn Jürg Schlup nun hier noch über den ach so enormen administrativen Aufwand der Ärzte jammert, ist das nur noch lächerlich. (Künzli 2020, Blick)

Der Leserbrief bezieht sich auf ein Interview mit dem Präsidenten der Verbindung der Schweizer Ärztinnen und Ärzte FMH Jürg Schlup (Schlup/Blum/Studer 2020), in dem dieser die »administrative Belastung für die Ärzteschaft« bei der Meldung von COVID-19-Fällen als »riesig« bezeichnet. Im Gegensatz zu ihm vertritt die Verfassende des Leserbriefs den Standpunkt *Das Verhältnis von Aufwand und Ertrag bei der Meldung von Erkrankungen an COVID-19 bietet der Ärzteschaft keinen Anlass zum Jammern. *Die sehr knappe Erzählung einer eigenen Erfahrung etabliert eine explizite Prämisse für diesen Standpunkt. Man könnte sie mit den Worten zusammenfassen: *Das Verhältnis von Aufwand und Ertrag bei der Meldung meiner Erkrankung an COVID-19 bot meinem Arzt keinen Anlass zum Jammern. *Dass die Erzählung als explizite Prämisse für den im letzten Satz enthaltenen Standpunkt dient, erkennt man daran, dass man die Erzählung und den Standpunkt mit *also *verbinden kann: *Mein Arzt erhält … Also ist es nur noch lächerlich, wenn Jürg Schlup …*

Außer der Rolle einer expliziten Prämisse für den expliziten oder impliziten Standpunkt übernimmt die Erzählung im untersuchten Korpus oft die Rolle einer expliziten Unterprämisse für eine (explizite oder implizite) Prämisse für den (expliziten oder impliziten) Standpunkt. Illustrieren lässt sich dieser Fall mit folgendem Leserbrief. Um die Darstellung zu vereinfachen, handelt es sich wiederum um einen sehr kurzen Brief:

(5) Meine Wahrnehmung von Corona in München (»Münchner unter sich« vom 15. April) und vom Viktualienmarkt: Auch ich war noch nie so oft am Viktualienmarkt wie im Augenblick, obwohl ich am Marienplatz arbeite. Es ist ruhig, fast beschaulich, man hört tatsächlich fast nur die bayerische Mundart, man kann mit den Standlbesitzern ratschen. Wir entschleunigen gerade. Es gibt trotz allem immer auch eine positive Seite, das muss man genießen. (Naglazas 2020, SZ)

Die neuen Eindrücke vom Viktualienmarkt, von denen hier erzählt wird, begründen eine explizite Prämisse, die im vorletzten Satz genannt wird: *Wir entschleunigen gerade.* Diese Aussage wiederum begründet den expliziten Standpunkt, der im letzten Satz zu finden ist: *Es gibt trotz allem …* Auch hier bestätigt dies der oben genannte Test mit *also. *Verbindet man nämlich die letzten beiden Sätze und die Erzählung versuchsweise mit *also*, entsteht eine sinnvolle Aussage: *Auch ich … Es ist ruhig, fast beschaulich … Also entschleunigen wir gerade. Also gibt es trotz allem immer auch eine positive Seite, die man genießen muss.*

Interessanterweise ist unter den ausgewerteten Leserbriefen kein einziger, in dem die Erzählung zweifelsfrei als Konklusion oder aber als bloße Erläuterung oder ausführlichere Wiederholung einer expliziten Prämisse oder Konklusion zu beschreiben wäre.

### Schema des narrativen Arguments und Standpunkt der Argumentation

Rekonstruiert man das narrative Argument der Leserbriefe und ordnet es dabei einem der gängigen Argumentationsschemata zu, zeigt sich, dass ein Argumentationsschema unangefochten dominiert: das des Arguments durch Induktion (vgl. dazu etwa Hastings 1962, S. 25–35; Kienpointner 1992, S. 365–372). Bei einem Argument durch Induktion wird von einem oder mehreren Beispielen auf einen Typ geschlossen, auf etwas Allgemeineres, das von dem oder den Beispielen repräsentiert wird. Man kann das Schema des Arguments durch Induktion so angeben (vgl. Schröter/Thome 2020, S. 297; Schröter 2021, S. 38):

1. Prämisse: Wenn etwas für ein oder mehrere repräsentative Exemplare eines Typs gilt, gilt es auch für den Typ.

2. Prämisse: A_1_, A_2_, A_3_ ... A_n_ sind die repräsentativen Exemplare, B ist der Typ.

3. Prämisse: Für A_1_, A_2_, A_3_ ... A_n_ gilt C.

-----------

Konklusion: Für B gilt C.

Auf dieses Schema lässt sich beispielsweise das narrative Argument zurückführen, aus dem der nächste Leserbrief besteht:

(6) Tatsache ist, dass DHL schon lange vor der Corona-Krise eine Zumutung war (»Die Zustellung wird zur Zumutung« vom 29. April). An meinem Gartentor hängt seit über einem Jahr ein großes Schild: »Pakete und Sendungen bitte einfach übers Tor werfen.« Eine Schelle hab ich auch. Die wird aber von DHL nicht benutzt: ein gelber Zettel geht schneller. Schon vor Weihnachten war das so. Da war dann das angeführte Postamt in der Savitsstraße einfach geschlossen. Ja: »Heute geschlossen« – mitten in der Woche. Am nächsten Tag wieder. Und am Montag drauf eine Schlage [sic] von 20 Personen – Wartezeit circa eine Stunde. Kurz drauf wieder ein gelber Zettel – aber die Sendung ließ sich auf dem Postamt nicht finden. Ich bin inzwischen bei Online-Bestellungen dazu übergegangen, ausdrücklich zu vermerken: Auf keinen Fall mit DHL. Oder ich fahre wie früher einfach mit dem Radl in die nächste Buchhandlung und kaufe dort ein. Das macht richtig Spaß. (Jacobi 2020, SZ)

Das Argument lässt sich so rekonstruieren:

1. Prämisse: Wenn etwas für ein oder mehrere repräsentative Exemplare eines Typs gilt, gilt es auch für den Typ.

2. Prämisse: Das Verhalten DHLs, das ich bei der Zustellung und Abholung eines Pakets erlebt habe, ist das repräsentative Exemplar, das Verhalten DHLs seinen Kunden gegenüber ist der Typ.

3. Prämisse: Das Verhalten DHLs, das ich schon vor der COVID-19-Krise bei der Zustellung und Abholung eines Pakets erlebt habe, war eine Zumutung.

-----------

Konklusion: Das Verhalten DHLs seinen Kunden gegenüber war schon vor der COVID-19-Krise eine Zumutung.

Die dritte Prämisse wird narrativ etabliert, indem verschiedene persönliche Erlebnisse mit DHL vor der COVID-19-Krise mitsamt den Reaktionen darauf chronologisch dargestellt werden. Dabei verstärken mehrere Formulierungen den Gegensatz zwischen dem Erwartbaren (Zustellung der Pakete durch Ablegen oder Klingeln, Öffnung der Poststelle zu den angegebenen Zeiten usw.) und dem tatsächlich Eingetretenen (Abholeinladung, Schließung der Poststelle usw.), wobei der Gegensatz mehrfach mit einem Gedankenstrich hervorgehoben wird. Aus der wiederholten Enttäuschung der Erwartung und der darauffolgenden Verhaltensänderung des Leserbriefautors ergibt sich, dass er das geschilderte Verhalten von DHL ihm gegenüber als *Zumutung *beurteilt.

Mehrere narrative Argumente des Korpus lassen sich darüber hinaus dem Schema des Arguments aus der Autorität zuordnen (vgl. exemplarisch Hastings 1962, S. 126–138; Kienpointner 1992, S. 393–402), das man auch als wichtigsten Subtyp des Arguments über die Eigenschaft einer These beschreiben kann. Bei diesem Argumentationsschema wird aus der Eigenschaft einer These (etwa der Eigenschaft, von einer Autorität vertreten zu werden) darauf geschlossen, dass die These stimmt. Das Schema des Arguments aus der Autorität kann man so angeben (vgl. Schröter/Thome 2020, S. 297; Schröter 2021, S. 38):

1. Prämisse: Wenn eine Autorität (z. B. ein Experte oder Zeuge) der Ansicht ist, dass etwas für einen Gegenstand ihres Kompetenzbereichs gilt, gilt es auch für den Gegenstand ihres Kompetenzbereichs.

2. Prämisse: A ist die Autorität, B ist der Gegenstand ihres Kompetenzbereichs.

3. Prämisse: A ist der Ansicht, dass für B C gilt.

----------

Konklusion: Für B gilt C.

Dieses Schema kann man z. B. in dem Argument erkennen, das im nächsten Zitat enthalten ist und wiederum den ganzen Leserbrief ausmacht:

(7) Es soll jetzt keiner sagen, man hätte sich doch ein Fettpolster anlegen sollen, um diese Zeit durchhalten zu können. Ich bin seit über zwei Jahren selbständig. In dieser Zeit habe ich mir und meiner Frau nie Lohn ausbezahlt und alles in die Firma gesteckt. Jetzt, in diesem Jahr, wo es anfängt lukrativ zu werden, kommt so eine Katastrophe. (Lendi 2020, Blick)

Man kann das Argument wie folgt rekonstruieren:

1. Prämisse: Wenn eine Autorität (z. B. ein Experte oder Zeuge) der Ansicht ist, dass etwas für einen Gegenstand ihres Kompetenzbereichs gilt, gilt es auch für den Gegenstand ihres Kompetenzbereichs.

2. Prämisse: Ich bin die Autorität, unternehmerische Selbstständigkeit ist der Gegenstand meines Kompetenzbereichs.

3. Prämisse: Ich bin der Ansicht, dass bei unternehmerischer Selbstständigkeit nicht zwingend finanzielle Rücklagen für die COVID-19-Krise gebildet werden konnten.

----------

Konklusion: Bei unternehmerischer Selbstständigkeit konnten nicht zwingend finanzielle Rücklagen für die COVID-19-Krise gebildet werden.

Allerdings lässt sich dieses Argument – wie auch alle anderen narrativen Argumente des Korpus, die man als Argumente aus der Autorität auffassen kann –, zugleich als Argument durch Induktion rekonstruieren. Die Doppeldeutigkeit ist nicht auf eine Unschärfe der Analysekategorien, sondern auf Eigenschaften der hier analysierten narrativen Argumente zurückzuführen: Bei allen narrativen Argumenten, die sich als Argumente aus der Autorität verstehen lassen, meldet sich einerseits eine Person zu Wort, die besonders qualifiziert ist (die z. B. einen Berufsstand vertritt oder eine Örtlichkeit besonders gut kennt) und deshalb als Autorität gelten kann. Andererseits wird immer von individuell-partikularen Erlebnissen und Erfahrungen erzählt, die verallgemeinerbar sind.

Nur in Ausnahmefällen ist das narrative Argument der untersuchten Leserbriefe als Aktualisierung eines dritten Argumentationsschemas beschreibbar: als Aktualisierung des Arguments über ein Verhältnis von Handlung und Qualität (für ein zumindest ähnliches Argumentationsschema vgl. Hastings 1962, S. 36–45). Dessen Schema lautet (vgl. Schröter/Thome 2020, S. 294; Schröter 2021, S. 33):

1. Prämisse: Wenn jemand eine charakteristische Handlung vollzieht, weist sie/er auch die entsprechende Qualität auf.

2. Prämisse: A ist die charakteristische Handlung, B ist die entsprechende Qualität.

3. Prämisse: C vollzieht A.

-----------

Konklusion: C weist B auf.

Dieses Schema kann man in folgendem knappen Leserbrief entdecken:

(8) Ich wurde letzten Donnerstag an meiner Schulter operiert im Spital Siloah in Gümligen. Trotz der schwierigen Zeiten war das Pflege- und Ärzte-Team immer sehr freundlich und hilfsbereit und aufgestellt. Alle haben versucht, den Aufenthalt so sicher und normal wie möglich zu gestalten. Ein herzliches Dankeschön an alle, die in diesen Tagen ihr Bestes geben für uns alle! Respekt! (Specchia 2020, Blick)

Man kann das Argument folgendermaßen rekonstruieren:

1. Prämisse: Wenn jemand eine charakteristische Handlung vollzieht, weist sie/er auch die entsprechende Qualität auf.

2. Prämisse: Während der COVID-19-Pandemie das Beste für die Patienten zu geben, ist die charakteristische Handlung, Dank und Respekt zu verdienen, die entsprechende Qualität.

3. Prämisse: Das medizinische Personal im Spital Siloah und evtl. auch andernorts gibt während der COVID-19-Pandemie das Beste für die Patienten.

-----------

Konklusion: Das medizinische Personal im Spital Siloah und evtl. auch andernorts verdient Dank und Respekt.

Wie man nach diesem und den vorausgehenden Beispielen bereits erahnen kann, stützt das narrative Argument in den hier betrachteten Leserbriefen stets einen evaluativen oder evaluativ-präskriptiven Standpunkt. Evaluative Standpunkte sind solche, die eine positive oder negative Bewertung enthalten. Präskriptive Standpunkte sind demgegenüber solche, die besagen, was getan oder nicht getan werden sollte. Wie sich in den obigen Beispieldiskussionen abgezeichnet hat, besteht der vertretene Standpunkt oftmals darin, dass ein bestimmter Umgang mit COVID-19 bzw. mit der dadurch verursachten Sondersituation oder eine Folge dieses Umgangs abgelehnt oder befürwortet wird. Solche Standpunkte liegen am Übergang zwischen evaluativen und präskriptiven Standpunkten. Die befürwortenden Standpunkte sind auffälligerweise gegenüber den ablehnenden deutlich in der Minderzahl. Erstere sind vor allem in den Schweizer Leserbriefen zu finden.

### Besonderheiten der Formulierung

Besondere Formulierungen lassen sich sowohl bei den Standpunkten als auch bei den Narrationen der untersuchten Leserbriefe feststellen.

So werden die expliziten Standpunkte häufig nicht direkt, sondern mit rhetorischen Fragen formuliert. Besonders oft ist dies in den beiden Qualitätszeitungen zu beobachten. Auf das häufige Vorkommen von rhetorischen Fragen in Leserbriefen hat bereits Ulla Fix (2012, S. 153–155) hingewiesen. Solche rhetorischen Fragen kann man als indirekte Sprechakte auffassen (vgl. Meibauer 1986, S. 8, 163–170, 183–185; Schröter 2021, S. 27). Als indirekter Sprechakt, mit dem der Standpunkt ausgedrückt wird, fungiert eine rhetorische Frage z. B. im folgenden Zitat:

(9) Am 8. Februar wurde bei der Einreise in Namibia meine Körpertemperatur gemessen und ich musste einen Fragebogen unterschreiben. Bei der Rückreise am 8. März über/in Amsterdam wurden wir weder gesundheitlich befragt noch wurde die Temperatur gemessen, als wenn es keine Corona-Pandemie geben würde. Auch Asiaten mit Atemschutzmasken wurden nicht kontrolliert. Weshalb lässt die EU die Flugpassagiere gesundheitlich unkontrolliert nach Europa? (Frey 2020, B.Z.)

Am Ende des Zitats werden die Entscheidungstragenden der EU nach ihren Gründen oder Argumenten dafür gefragt, dass sie Flugreisende ohne Kontrolle auf COVID-19 in die EU einreisen lassen. Daraus lässt sich erschließen, dass dem Schreibenden keine ausreichenden Gründe dafür einfallen und er folglich den Standpunkt vertritt: *Flugpassagiere ohne Kontrolle auf COVID-19 in die EU zu lassen, ist abzulehnen. *Weitere Beispiele für rhetorische Fragen, die Standpunkte formulieren, sind:

- »Was soll das?« (Helmut 2020, Blick),
- »Wollen wir das jetzt wirklich so schnell wieder kaputt machen […]?« (Frei 2020, SZ),
- »Geht es nicht gerade darum, die Risikopatienten zu schützen? Warum wird immer nur über ältere Menschen gesprochen? […] Kann es sein, dass Kinder anhand von Jahrgängen wieder zurück in die Schule geschickt werden? Warum lässt man nicht alle Kinder zurück, die keinen Kontakt zu Risiko behafteten Menschen haben, und findet für die anderen eine Corona-Lösung? […] Wäre es nicht viel sinnvoller, Lösungen zu suchen, die Familien mit Angehörigen einer Risikogruppe helfen, sich zu schützen? […] Und den Rest der Kinder ganz normal in den Kindergarten, die Schule gehen zu lassen? […] warum nicht Lösungen rund um den Schutz von Risikogruppen suchen statt alle zwangsverpflichten?« (Rico Castillo 2020, SZ),
- »Darf es sein, dass immer die Kleinstverdienenden den Kürzeren ziehen, diejenigen, die kein Geld für Anwaltskosten haben?« (Kunz 2020, TA),
- »Ist das richtig? Wo ist da die Wertschätzung?« (Merz Iten 2020, TA) etc.

Wie die Beispiele zeigen, wird nicht immer nach Gründen, sondern auch nach Absichten und Bewertungen gefragt, über die es möglich ist, den Standpunkt der/des Schreibenden zu erschließen. Zudem wird deutlich, dass ein Standpunkt auch mit mehreren, ja sogar mit einer ganzen Serie rhetorischer Fragen ausgedrückt werden kann.

Der funktionale Mehrwert solcher rhetorischen Fragen lässt sich darin sehen, dass sie die Lesenden dazu bringen, selbstständig zum Standpunkt des jeweiligen Leserbriefs zu finden. Im Vergleich zur Formulierung von Standpunkten mit Aussagesätzen ist deren Formulierung mit Fragesätzen behutsamer, eröffnet den Lesenden mehr Freiräume und aktiviert sie kognitiv stärker (ähnlich Meibauer 1986, S. 169–170). Dies könnte helfen, Widerspruch gegenüber dem jeweiligen Standpunkt zu vermeiden oder zu überwinden.

Auch die Narrationen der Leserbriefe des Korpus weisen Formulierungsbesonderheiten auf. Die obigen Beispiele haben bereits angedeutet, dass deren stilistische Vielfalt beachtlich ist. So finden sich beispielsweise in einigen Narrationen humorvolle Interjektionen, in anderen betont umgangssprachlich-saloppe oder dialektale Ausdrücke, in wieder anderen Wiederholungsfiguren wie Parallelismen und Anaphern, aber auch telegrammartige Ellipsen bzw. Auslassungen kommen vor. So kann im Folgenden nur auf die häufigsten Formulierungsbesonderheiten der Narrationen eingegangen werden. Diese lassen sich zu zwei funktional bestimmten Gruppen zusammenfassen. Zum einen finden sich wiederkehrende formale Besonderheiten, die eine bestimmte Bewertung des erzählten Geschehens durch die Lesenden nahelegen. In diese erste Gruppe gehören Ausdrücke mit intensivierender Semantik (Gradpartikel, entsprechende Adjektive), die in vielen Narrationen vorkommen. Sie wie auch die weiteren genannten Formulierungsbesonderheiten finden sich oft mehrfach in derselben Erzählung, und bei ihnen wie auch bei den weiteren genannten Formulierungsbesonderheiten geht die Hervorhebung in den Belegzitaten auf mich zurück:

- »*sehr* gut« (Luginbühl 2020, Blick),
- »*ständig* […] angesprochen« (Becker 2020, B.Z.),
- »*starke* Symptome« (Pöppel 2020, B.Z.),
- »*gar* nicht« (Rebschläger 2020, B.Z.),
- »*ganz* gelassen« (Frei 2020, SZ),
- »*wesentlich* mehr« (Lehner 2020, SZ),
- »*richtig* gerne« (Lepski 2020, SZ),
- »einem *wirklich* schönen Biotop« (Will 2020, SZ) usw.

Zur ersten Gruppe zählen ebenfalls Ausdrücke mit verabsolutierender Semantik (absolute Adverbien, indefinite Artikelwörter/Pronomen, superlativische Adjektive):

- *»überall«* (Helmut 2020, Blick),
- »*alles *in die Firma gesteckt« (Lendi 2020, Blick),
- »*jederzeit* erreichbar« (Luginbühl 2020, Blick),
- »bei *allen *Telefonnummern nicht« (Sander 2020, B.Z.),
- »mit *besten* Rahmenbedingungen« (Lepski 2020, SZ),
- »*nie* gekümmert« (Richter 2020, SZ),
- »*Jeder* ist bemüht« (Fichmann 2020, TA),
- »meine *schönste* und *wichtigste* soziale Aktivität« (Zünd 2020, TA) u. a.

Ausdrücke mit intensivierender und verabsolutierender Semantik (die Bubenhofer/Spieß 2012, S. 97, vgl. 95–99, im Anschluss an Os 1989 als »Intensivierer« bezeichnen) dienen in den Narrationen vor allem dazu, das Geschehen, von dem erzählt wird, zuzuspitzen oder sogar zu übertreiben: etwas war nicht *gut*, sondern *sehr gut*, jemand wurde nicht* oft*, sondern* ständig angesprochen*, etwas ist nicht *vielerorts*, sondern *überall *der Fall, jemand hat nicht *viel*, sondern *alles in die Firma gesteckt* usw. Dadurch kann der Kontrast zwischen dem Erwarteten bzw. Erwartbaren und dem tatsächlich Eingetretenen verstärkt, die Erzählwürdigkeit des Geschehens erhöht und zugleich eine klar positive oder negative Bewertung des Geschehens nahegelegt werden.

Zu den rekurrenten Formulierungsbesonderheiten der Narrationen, die eine bestimmte Bewertung des erzählten Geschehens durch die Lesenden anregen, gehören sodann Wörter (Substantive, Verben, Adjektive) entweder mit primär evaluativer Bedeutung oder mit primär denotativer und zusätzlich evaluativer Bedeutung (zum Konzept der evaluativen Bedeutung vgl. vor allem Hermanns 2012, S. 159). Zu den Wörtern mit primär evaluativer Bedeutung, die hauptsächlich eine Bewertung zum Ausdruck bringen, gehören beispielsweise:

- »Riesentamtam« (Keller 2020, Blick),
- »Katastrophe« (Lendi 2020, Blick),
- »Unannehmlichkeiten« (Richter 2020, SZ),
- »wunderbare« (Will 2020, SZ),
- »Win-Win-Situation« (Wolf 2020, SZ),
- »herrlich« (Bosshard 2020, TA),
- »wunderbar« (Fichmann 2020, TA),
- »Problemfall« (Spichale 2020, TA) usf.

Wörter mit primär denotativer und zusätzlich evaluativer Bedeutung sind Wörter, die vorrangig etwas bezeichnen und dies zugleich bewerten. Die folgenden Beispiele für solche Wörter werden der besseren Verständlichkeit halber im weiteren Kotext zitiert:

- »Der Zugang zum Wasser ist *verrammelt* und *verriegelt*« (Helmut 2020, Blick),
- »Mein Einkommen ist […] *abgestürzt*« (Gregoror 2020, B.Z.),
- »die Stadt […] hat […] uns […] mit *Wischi-Waschi-Worten ruhigzustellen* versucht« (Richter 2020, SZ),
- »dass […] am Parlament *vorbeiregiert* wird« (Rogler 2020, SZ),
- »Meinen Kunden biete ich […] individuelle,* kreative* und *nachhaltige *Lösungen an« (Wolf 2020, SZ),
- »Dass meiner Mutter und mir so etwas *zugemutet *wird« (Boss 2020, TA),
- »ich […] muss mich *ausgrenzen* lassen« (Spichale 2020, TA),
- »dass der Bundesrat […] die Demokratie und den Föderalismus *ausgeschaltet *[…] hat« (Stadelmann 2020, TA) u. a.

Wörter mit vorrangig evaluativer Bedeutung bewerten die erzählten Zustände ganz direkt: diese sind ein *Riesentamtam*, eine *Katastrophe* usw. Wörter mit ergänzender evaluativer Bedeutung unterlegen dem erzählten Geschehen hingegen *en passant* eine bestimmte Bewertung: *Der Zugang zum Wasser ist* nicht *gesperrt,* sondern *verrammelt *und *verriegelt*, das *Einkommen ist* nicht gesunken, sondern *abgestürzt *usw. Wörter mit zusätzlicher evaluativer Bedeutung können mithin nach und nach wertende Bedeutungsaspekte in die Erzählung einbringen, die sich im Laufe der Darstellung zu einer stark ausgeprägten, geradezu unumstößlichen Bewertung des Geschehens verdichten.

Noch eine weitere wiederkehrende Formulierungsbesonderheit, die eine bestimmte Bewertung des erzählten Geschehens nahelegt, soll erwähnt werden: die Beschreibung der Gefühle der/des Schreibenden und Erzählenden mit Substantiven, Verben und Adjektiven, die affektiv-emotionale Zustände, Wirkungen bzw. Reaktionen bezeichnen:

- »Das ist *belastend* für mich« (Becker 2020, B.Z.),
- »Wir sind *fassungslos*« (Schmidt 2020, B.Z.),
- »*Ohnmacht, Unverstandensein* und auch *Hilflosigkeit fühle* ich« (Rogler 2020, SZ),
- »Aber so […] *tut* mir das in der Seele *weh*« (Will 2020, SZ),
- »Dass […], macht mich *wütend* und *traurig* zugleich« (Boss 2020, TA),
- »Dass […], macht mich *traurig *und auch etwas *ratlos*« (Kunz 2020, TA),
- »Was mich […] *tief verletzt*« (Merz Iten 2020, TA),
- »Die Vorstellung […] macht mich *fertig*« (Zünd 2020, TA) usw.

Indem die Schreibenden verbalisieren, welch starke Gefühle das Geschilderte bei ihnen ausgelöst hat, regen sie die Lesenden dazu an, empathisch zu sein, sich in sie hineinzuversetzen und ihnen nachzufühlen. Auf diese Weise können Gefühlsbeschreibungen die Lesenden dazu bringen, zunächst versuchsweise und dann vielleicht sogar dauerhaft die Perspektive der Erzählenden einschließlich der Bewertung des Geschilderten zu übernehmen.

Die zweite Gruppe von formalen Besonderheiten, die in den argumentativen Narrationen der untersuchten Leserbriefe vielfach belegt sind, dient insgesamt der Plausibilisierung des erzählten Geschehens, verstärkt also den Faktizitätsanspruch der Erzählung. In diese Gruppe fallen zum einen eindeutige Ortsangaben wie z. B.:

- »im Spital Siloah in Gümligen« (Specchia 2020, Blick),
- »Ecke Holzhauser Straße« (Rebschläger 2020, B.Z.),
- »beim Bürgeramt Lichtenberg« (Sander 2020, B.Z.),
- »an der Isar (Stauwehr Oberföhring)« (Frei 2020, SZ),
- »das angeführte Postamt in der Savitsstraße« (Jacobi 2020, SZ),
- »Flughafen Bangkok. […] Flughafen Amsterdam. […] Flughafen Frankfurt. […] Flughafen München. […] Landshut« (Langwieser 2020, SZ),
- »in Freiburg« (Lepski 2020, SZ),
- »auf dem Uetliberg« (Suter 2020, TA) etc.

Zum anderen gehören Zeitangaben dazu, die oft sehr genau sind, beispielsweise:

- »letzten Donnerstag« (Specchia 2020, Blick),
- »Am 8. Februar […] am 8. März« (Frey 2020, B.Z.),
- »um 05.20 Uhr« (Rebschläger 2020, B.Z.),
- »Seit Ende Mai […] im April« (Sander 2020, B.Z.),
- »Beim Abendlauf (21 Uhr)« (Frei 2020, SZ),
- »Um 14.19 Uhr« (Langwieser 2020, SZ),
- »Um 23.30 Uhr« (Mayer-Schwender [sic]/Schwendner 2020, SZ),
- »vom 6‑Uhr-Geläut« (Bosshard 2020, TA) usf.

Solche Orts- und Zeitangaben kennzeichnen die Umstände der erzählten Zustände bzw. Zustandsveränderungen soweit, dass sie für die Lesenden überprüfbar sind – oder jedenfalls vermitteln sie den Eindruck, dass sie überprüfbar wären. Orts- und Zeitangaben signalisieren somit eine quasi-protokollarische Exaktheit, die die Erzählung glaubwürdiger macht und Zweifel an der Faktizität sowie womöglich auch an der Repräsentativität bzw. Typik des Erzählten ausräumen kann.

## Zusammenfassung und Deutung

Für diesen Beitrag wurden gut 50 Leserbriefe analysiert, in denen argumentiert wird, indem (unter anderem) erzählt wird. Die Leserbriefe stammen aus vier deutschsprachigen Tageszeitungen, haben alle einen Bezug zur Infektionskrankheit COVID-19, setzen aber innerhalb dieses thematischen Rahmens unterschiedliche Schwerpunkte. Dass es nicht schwer war, solche Leserzuschriften in genügender Anzahl zu finden, bestätigt, wovon die Analyse ausgegangen ist: dass nämlich viele zeitgenössische politische Leserbriefe erzählende Abschnitte enthalten und dass diese Abschnitte häufig der Argumentation dienen.

Wie werden Erzählungen in zeitgenössischen politischen Leserbriefen zur Argumentation verwendet? Die Analyse hat ergeben, dass in den hier gewählten Leserbriefen normalerweise genau eine Erzählung vorkommt, in der die/der Schreibende persönliche Erlebnisse und Erfahrungen schildert. Diese Narration variiert nicht nur thematisch, sondern auch formal: Sie kann sich in einem kurzen oder sehr kurzen, aber auch in einem ausführlichen oder sehr ausführlichen Leserbrief befinden. Dementsprechend kann es sich um eine kurze oder sehr kurze Mikroerzählung handeln, jedoch ebenso um eine längere, wendungs- und detailreiche Erzählung. Die Narration wird normalerweise von anderen, oft rein argumentierenden Textteilen begleitet, einzelne Leserbriefe bestehen allerdings aus nichts anderem als der Narration. Da in der Textsorte *Leserbrief* typischerweise ein Standpunkt vertreten wird, lässt sich die Narration in solchen Fällen als explizite Prämisse eines argumentativen Schlusses auffassen, bei dem die Konklusion, die zugleich den Standpunkt bildet, implizit bleibt. Üblicher ist es jedoch, dass die Narration eine explizite Prämisse für den expliziten Standpunkt etabliert. In diesen Fällen wird der Standpunkt ausdrücklich formuliert und mit der Erzählung begründet. Oftmals ist die Narration auch als explizite Unterprämisse für eine Prämisse für den Standpunkt zu beschreiben. Dann gibt es den Standpunkt, dieser wird begründet, und die Begründung wird wiederum mit der Erzählung begründet. Entweder der Standpunkt oder aber die Begründung können dabei implizit bleiben, lassen sich in diesem Fall aber aus dem Kontext erschließen. Bei den argumentativen Schlüssen, zu denen die Narration beiträgt, den narrativen Argumenten, handelt es sich großmehrheitlich um Argumente durch Induktion. Hin und wieder sind die narrativen Argumente aber ebenso als Argumente aus der Autorität analysierbar. Die narrativen Argumente stützen stets einen evaluativen oder evaluativ-präskriptiven Standpunkt. Sowohl die Standpunkte als auch die Narrationen weisen musterhafte Formulierungsbesonderheiten auf. Die expliziten Standpunkte werden vielfach mit rhetorischen Fragen formuliert. Die Narrationen enthalten demgegenüber auffällig viele Ausdrücke mit intensivierender Semantik, Ausdrücke mit verabsolutierender Semantik, Wörter entweder mit primär evaluativer Bedeutung oder mit primär denotativer und zusätzlich evaluativer Bedeutung sowie Beschreibungen der Gefühle der/des Schreibenden. Diese formalen Besonderheiten vermitteln eine bestimmte Bewertung des erzählten Geschehens. Weitere Formulierungsbesonderheiten, die oft vorkommen, sind eindeutige Ortsangaben und genaue Zeitangaben. Sie unterstützen den Faktizitätsanspruch der Erzählung.

Und warum werden Erzählungen in zeitgenössischen politischen Leserbriefen nun zur Argumentation verwendet? Nach den zuletzt zusammengefassten Ergebnissen lässt sich die Frage so konkretisieren: Welcher funktionale Mehrwert ergibt sich für die Schreibenden daraus, die Prämisse eines Arguments durch Induktion in einer Argumentation mit evaluativem Standpunkt als Erzählung zu formulieren? Bei der Suche nach Antworten ist zunächst daran zu erinnern, dass bei solchen Argumenten von einem oder mehreren Beispielen auf einen Typ geschlossen wird, auf etwas Allgemeineres, das von dem oder den Beispielen repräsentiert wird. Die Erzählung der untersuchten Leserbriefe liefert jeweils das Beispiel. Gegenüber einer nicht-narrativen Formulierung des Beispiels hat dessen narrative Entfaltung zunächst naheliegenderweise den Vorteil, dass sie plastischer und lebendiger sein kann. Die Lesenden können sich das Beispiel somit besser vorstellen. Die narrative Entfaltung des Beispiels hat zudem den Vorzug, dass sie mit Kontextinformationen und Details angereichert werden kann (ähnlich z. B. Tindale 2017, S. 28). Die Lesenden können das Beispiel dadurch eher als wirklichkeitsgetreues, den Tatsachen entsprechendes annehmen (ähnlich z. B. Girnth/Burggraf 2019, S. 110). Der wichtigste Vorteil der narrativen Entfaltung des Beispiels aber scheint zu sein, dass diese die Möglichkeit bietet, mit dem beispielhaften Geschehen sukzessive auch dessen Bewertung zu vermitteln – und zwar auf eine Weise, die allfälligen Widerspruch der Lesenden verhindern oder überwinden kann. Die Erzählung lädt dazu ein, sich in den/die Erzählende hineinzuversetzen und das Geschehen aus ihrer/seiner Perspektive zu imaginieren (ähnlich z. B. Weidacher 2018, S. 315; Girnth/Burggraf 2019, S. 111). Die Bewertung des Geschehens wird den Lesenden im Laufe der Lektüre einerseits durch die nacherlebten Ereignisse und andererseits durch die verschiedenen herausgearbeiteten sprachlichen Besonderheiten nahegelegt. In vielen Fällen scheint eine erzählte Prämisse also eine besonders große Chance zu haben, den Status der Unstrittigkeit bei den Lesenden zu erlangen – und zwar gerade mit Blick auf die enthaltene Bewertung. Die Prämisse eines Arguments durch Induktion zu erzählen, hat für die Schreibenden folglich den Mehrwert, dass sie eine Argumentation, und zwar insbesondere eine mit evaluativem Standpunkt, überzeugender machen kann.

Die Frage nach dem funktionalen Mehrwert lässt sich aber natürlich nicht nur mit Blick auf die einzelnen Schreibenden stellen. Gibt es auch einen gesellschaftlich-kulturellen Mehrwert narrativer Argumente durch Induktion in Argumentationen mit evaluativem Standpunkt, einen Mehrwert insbesondere für politische Auseinandersetzungen? Einen solchen Mehrwert könnte man darin erkennen, dass narrative Argumente durch Induktion auf unkomplizierte Weise die soziale Mikroebene mit der gesellschaftlichen Makroebene verbinden können. Die Erzählungen schildern mindestens zwei zeitlich aufeinanderfolgende Zustände derselben menschlichen oder anthropomorphen Entität, sie schildern also *per definitionem* Individuelles, Partikulares, Konkretes. Mit Argumenten aus der Induktion wird versucht, die enthaltenen Bewertungen auf Abstrakteres, Kollektives, Soziales zu übertragen. Dadurch können narrative Argumente durch Induktion das medial vermittelte, oft eher abstrakte Bild aktueller gesellschaftlich-politischer Problemlagen um konkrete Schnappschüsse aus individuellen Lebenswirklichkeiten erweitern. Vor diesem Hintergrund sind narrative Argumente durch Induktion in gesellschaftlich-politischen Auseinandersetzungen nicht per se als zweitklassige oder gar defizitäre Argumente zu beurteilen – etwa nach dem Motto: *Wer nicht gut argumentieren kann, erzählt*. Es handelt sich vielmehr um potenziell wertvolle, vielleicht sogar unersetzliche Beiträge zu diesen Diskussionen: *Wer gut argumentieren kann, erzählt auch*, wäre die zutreffendere Aussage. Zwar lässt sich im Alltag bisweilen eine wertende Entgegensetzung von politischer Argumentation und Narration beobachten, wenn z. B. behauptet wird, dass Argumentationen in deliberativen Prozessen Erzählungen überlegen seien oder dass Storytelling in politischen Kampagnen effektiver als Argumentieren sei. Nach den vorgestellten Ergebnissen sind solche Entgegensetzungen aber fragwürdig, weil sie verkennen, wie oft Argumentation in der politischen Kommunikation durch Narration realisiert wird.

»For the most part, people tell stories to do something – to complain, to boast, to inform, to alert, to tease, to explain or excuse or justify« *– or to present an argument*, könnte man das Diktum Emanuel Schegloffs (1997, S. 97), mit dem dieser sich auf Marjorie H. Goodwin bezieht, am Ende dieser Untersuchung ergänzen. Die Untersuchung hat das Spektrum der argumentativen Narrationen, die bislang in der Forschung diskutiert wurden, erweitert und dabei das Wissen um die möglichen Formen und Funktionen dieser Erzählungen vertieft. Je größer dieses Wissen wird, desto mehr drängt sich die Frage auf, ob argumentative Narrationen eigentlich einen Sonderfall bilden oder ob sie nicht vielmehr der Normalfall sind. Lassen sich möglicherweise die meisten Narrationen, und zwar Erzählungen in der politischen Kommunikation ebenso wie Alltags- und literarische Erzählungen, argumentativ verstehen, weil sie fast alle dazu einladen, aus dem Erzählten einen oder mehrere weiterreichende Schlüsse zu ziehen? Dass Letzteres für viele Romane gilt, ist bereits vorgeschlagen worden (vgl. vor allem Plumer 2015, S. 497–505). Inwiefern die genannte Hypothese tatsächlich haltbar ist, müssen weitere empirische Studien zu literarischen, aber gerade auch zu nicht-literarischen Erzählungen zeigen.
